# “Look at Yourself”: Teachers' Reflective Practices Toward Enjoyment in Primary School Physical Education

**DOI:** 10.1111/josh.70092

**Published:** 2025-10-07

**Authors:** Anoek Adank, Nina Bartelink, Steven Vos, Dave Van Kann

**Affiliations:** ^1^ School of Sport Studies Fontys University of Applied Sciences Eindhoven the Netherlands; ^2^ Department of Industrial Design Eindhoven University of Technology Eindhoven the Netherlands; ^3^ Department of Health Promotion Research Institute of Nutrition and Translational Research in Metabolism (NUTRIM), Maastricht University Maastricht the Netherlands

**Keywords:** enjoyment, physical education, primary school, professional development, reflection, teachers

## Abstract

**Background:**

Enjoyment is key in primary school physical education (PE), yet ensuring enjoyable PE experiences for all children is challenging. Reflection may help teachers improve lesson quality and foster PE enjoyment.

**Methods:**

This cross‐sectional study explored Dutch primary school teachers' perspectives on enjoyable PE and reflective practices. An online questionnaire was completed by 173 teachers (70.5% PE specialists, 29.5% generalists) who teach at least one PE lesson per week. Teacher type differences were analyzed using Chi‐square tests, Mann–Whitney *U* tests, and logistic regressions.

**Results:**

Perspectives on reflective practices were similar among teacher types. Generalists reported more teacher‐ and policy‐related barriers to providing enjoyable PE, while specialists cited child‐related challenges. Most teachers reflected internally, with limited use of written reflection or feedback from children and colleagues. Specialists used professional networks and peer feedback more. Time constraints and low prioritization were key barriers to reflection.

**Implications for School Health Policy:**

Schools should allocate time and promote reflection tools. Generalists could benefit from coaching and targeted professional development. For specialists, teacher education might foster reflective practices using video and child feedback to improve child outcomes like PE enjoyment.

**Conclusions:**

Tailored strategies are needed to strengthen reflection and improve PE quality to foster children's PE enjoyment.

## 1 Introduction

Sufficient physical activity (PA) and limited sedentary behavior (SB) are important for children's physical and mental health [1, 2, 3], as early PA behaviors predict lifelong activity patterns [4]. Physical education (PE) in primary schools plays a key role [5] in fostering children's development of skills, knowledge, and attitudes that promote lifelong participation in PA. Central to achieving these outcomes is fostering children's enjoyment of PE, which increases when PE is delivered in a need‐supportive environment. A need‐supportive environment is considered one that satisfies children's basic psychological needs for autonomy, competence, and relatedness. Such environments enhance children's engagement, motivation, and learning in PE [6], increase enjoyment of and motivation for PA [7], and help reduce SB outside the school context [8].

Delivering high‐quality primary school PE that is enjoyable for all children, however, is a challenging task, given the complexity of the PE environment. Teachers must respond to various factors, including differences among children in terms of skill levels, interests, characteristics, and learning needs. They must also account for external factors related to PE facilities and policy, such as the availability of facilities and equipment, the time allocated for PE lessons, institutional support, and opportunities for teachers' professional development [9, 10, 11, 12]. Addressing these challenges requires PE teachers to have professional, pedagogical, and didactic competencies [13, 14].

Reflection can help teachers develop and enhance these competencies. In the present study, reflection is defined as a systematic, cyclical process that involves evaluating past lessons, identifying areas for improvement, exploring alternative teaching practices, and implementing these strategies in future lessons [15, 16, 17]. PE lesson evaluations form a vital component of this reflection process, as teachers look back on their lessons by describing, explaining, and analyzing their teaching experiences [16]. Child feedback can enrich these evaluations, providing teachers with valuable insights into children's perspectives, preferences, and needs for PE enjoyment [18, 19, 20], while also fostering a stronger teacher‐pupil relationship by ensuring children feel heard [20, 21]. Additionally, collaborative reflection with colleagues further supports professional learning and development by providing alternative points of view, shared experiences, and constructive feedback that help identify and implement improved teaching practices [22, 23, 24, 25].

Previous PE‐related research has shown that reflective strategies, such as self‐evaluation questionnaires, written logbooks, video recordings, peer observations, and structured reflective discussions, can support (pre‐service) teachers' reflective processes and enhance their teaching effectiveness. The structured use of these reflection strategies supports (pre‐service) teachers' professional learning and development [26, 27, 28, 29]. It is therefore hypothesized that engaging in reflection enables PE teachers to identify areas for improvement and address them, ultimately improving teaching quality and fostering greater enjoyment of PE.

Despite its potential, reflection is not widely embedded in primary school PE practice. Teachers face barriers to engage in reflection, including a lack of perceived value, insufficient knowledge and skills, time constraints, and limited encouragement from school leadership [30]. Moreover, teachers rarely ask for feedback from their children [14], thereby underutilizing valuable sources of information for improving their teaching practice.

Given current PE goals, in which teachers play a leading role in ensuring PE enjoyment for every child, it is crucial to understand how teachers perceive the importance of creating enjoyable PE lessons and their perceived barriers to creating enjoyable PE lessons. Additionally, understanding the challenges teachers encounter in reflecting on their teaching is important, as reflection enables them to evaluate lesson quality and identify areas of improvement. These insights are prerequisites for supporting teachers' continuous professional development and designing effective strategies to enhance both their reflective practices and teaching competencies.

A better understanding of how primary school teachers reflect on their PE teaching can further inform such strategies, particularly in countries like the Netherlands, where primary school PE is taught by various “types” of teachers, that is, PE specialists, generalists, or a combination of both. Generalists complete a shorter training course to become qualified to provide PE at the primary level, while PE specialists follow a comprehensive PE teacher education program to teach PE across all educational levels [31]. PE specialists often teach primary school PE full‐time across multiple classes, whereas generalist teachers may deliver one or two PE lessons per week to their class, in addition to general classroom lessons. These different types of teachers may differ in the barriers they face in delivering enjoyable PE lessons and engaging in reflective practices, as well as in their perspectives on and actual practices of teaching and reflection. For example, a study by Truelove et al. [12] found that generalists reported barriers such as limited time, inadequate facilities and equipment, insufficient training, lack of knowledge, and low self‐efficacy in delivering primary school PE. PE specialists did not report these barriers.

To the best of our knowledge, no study has yet explored the perspectives of primary school teachers on creating enjoyable PE lessons, their use of reflective practices, and the differences between PE specialists and generalists in this area. Therefore, the aim of this study was to explore primary school teachers' perspectives on providing enjoyable PE lessons, including the barriers they perceive and their use of reflection to enhance teaching practices. This study also examined potential differences between PE specialists and generalists to inform the development of tailored strategies that support reflective practices, enhance teaching quality, and promote PE enjoyment among children.

## 2 Methods

### 2.1 Participants

The current study used a cross‐sectional design. Participants included both primary school generalists and PE specialists who teach at least one PE lesson per week. Dutch generalists have completed a four‐year teacher education program (240 European Credit Transfer and Accumulation System [ECTS]) to become primary school teachers and have followed a 40‐ECTS training course to become qualified to teach primary school PE. In contrast, Dutch PE specialists have completed a 240‐ECTS PE teacher education program qualifying to teach PE at primary, secondary, and tertiary levels [31].

Participants were recruited through professional associations for (PE) teachers, as well as national, regional, and local organizations that employ primary school (PE) teachers, via email and social media (LinkedIn). Recipients were encouraged to share the invitation with colleagues (generalists or PE specialists) teaching PE in primary schools. This approach aimed to ensure a nationally representative and diverse sample across regions and types of school. Recipients were informed about the study's purpose and methods and invited to complete an anonymous, one‐time online questionnaire. A reminder was sent 3 weeks later. Ethical consent was obtained by the Ethics Committee of Fontys University of Applied Sciences (reference number FCEO 24‐03 Adank). Written informed consent for data collection, processing, and analysis was obtained at the start of the questionnaire. As an inclusion criterion for participation, teachers had to teach at least one lesson per week. Only complete questionnaires were included in the analyses.

### 2.2 Instrumentation

The questionnaire developed for this study consisted of three sections: (i) demographics, (ii) perspectives on providing enjoyable PE lessons, and (iii) perspectives on reflective practices. Prior to these sections, participants were presented with a screening question to ensure they provided at least one PE lesson per week. The questionnaire was pilot tested with three PE teachers in April 2024. Based on their feedback, a few questions were revised to improve clarity. Data collection took place in May and June 2024. It took participants approximately 15 min to complete the questionnaire.

#### 2.2.1 Demographics

Demographic data on the teachers were collected, including age, gender, years of experience in primary school PE teaching, and whether they were PE specialists or generalists (teacher type).

### 2.3 Perspectives on Providing Enjoyable PE Lessons

To assess whether teachers value children's PE enjoyment and actively foster enjoyment during lessons, two single items on a 4‐point Likert scale (ranging from 1 = “agree” to 4 = “disagree”) were utilized. Additionally, a question was included to identify the key barriers teachers face in providing enjoyable PE lessons. Participants were asked to select up to five barriers from a provided list or choose the ‘other’ option to specify additional barriers. The listed options were based on barriers reported in previous research [9, 10, 11, 12], and grouped into four categories: (i) teacher‐related factors, such as PE‐specific knowledge and skills and self‐confidence in teaching PE (*n* = 7), (ii) child‐related factors, such as children's interest and motivation (*n* = 5), (iii) school facility factors, such as quality of the PE accommodation and available equipment (*n* = 8), and (iv) school policy factors, such as PE scheduling and PE lesson preparation time (*n* = 5).

### 2.4 Perspectives on Reflective Practices

To assess teachers' perspectives on reflective practices and willingness for professional growth, we selected four constructs, each consisting of four items (see Supporting Information), from the validated Reflective Practice Questionnaire by Priddis and Rogers, which initially includes 11 constructs, measured on a 6‐point Likert scale [32]. This questionnaire has been used in multiple studies across different contexts (e.g., education, nursing, psychology) and has demonstrated good validity and reliability [33, 34]. The selected constructs reflect key aspects of reflection, including self‐evaluation (construct “reflection on action”), self‐assessment (construct “self‐appraisal”), reflection with others (construct “reflection with colleagues”), and the desire to improve one's teaching practices (construct “desire for improvement”). In the current study, the scale reliability of these constructs, measured by Cronbach's alpha, ranged from fair to good, with values of 0.68, 0.80, 0.63, and 0.76, respectively. Additionally, to assess a construct examining teachers' openness to feedback from children for reflecting and improving their PE teaching, three single items (see Supporting Information) were adapted from Choy's Reflective Thinking Questionnaire [35]. All items were adjusted to the PE context and translated into Dutch. Responses were collected on a 4‐point Likert scale (1 = “agree” to 4 = “disagree”) to reduce and simplify interpretation for participants. In the current study, Cronbach's alpha for the construct “feedback from children” was 0.80.

To gain a deeper understanding of how teachers currently engage in reflective practices, participants were asked about the frequency, methods, and perceived barriers associated with their reflection on teaching PE. The frequency of engagement in reflection was measured using a 4‐point Likert scale, ranging from “(almost) always (1)” to “(almost) never (4).” To identify the reflective methods as well as applied lesson evaluation strategies, participants were asked to select one or more predefined strategies commonly recognized in prior research, such as “using a logbook” or “analysing recorded PE lessons.” [28, 29] They were also allowed to add their strategies.

To examine the reasons teachers do not engage in reflection, respondents selected one or more barriers from a list of commonly reported barriers, such as lack of time and insufficient support [36, 37]. Participants could also provide additional reasons in their own words.

## 3 Data Analysis

Data were analyzed using SPSS Statistics (version 28; Armonk, NY: IBM Corp). Descriptive statistics were conducted for demographic variables, including age, gender, years of PE teaching experience, and teacher type (generalist or PE specialist). Additionally, descriptive analyses were performed to examine teachers' value of and frequency in consciously providing enjoyable PE lessons, frequency of reflective practice, methods used for evaluating PE lessons and reflecting on PE teaching, as well as perceived barriers to reflection. To compare generalists and PE specialists regarding reflection methods and perceived barriers to reflection, Chi‐square tests were conducted.

For the perceived barriers to providing enjoyable PE lessons, the proportion of selected barriers within each category was calculated for each participant, relative to their total number of selected barriers, resulting in a score between 0 and 1 per category. The mean proportion score for each main category was calculated across all participants, reflecting the relative occurrence of each category. A score of 0 indicated no selection within this category, while a score of 1 indicated that all five selected barriers fell into this category. To assess overall differences in barrier selection across the four categories, a non‐parametric Friedman test was used on mean proportion scores. Unadjusted non‐parametric Mann–Whitney *U* tests were conducted to compare the mean proportion scores between generalists and PE specialists.

For constructs assessing perspectives on reflective practices, normality tests (Shapiro–Wilk tests) were conducted to evaluate the distribution of construct scores. As the assumption of homogeneity was violated for several constructs, responses were dichotomized: “agree” and “somewhat agree” were recoded as 1, and “somewhat disagree” and “disagree” as 0. Construct sum scores were then calculated by summing the binary responses per construct, resulting in scores ranging from 0 to 4. These scores were further dichotomized: a score of 1 was assigned if all four items within a construct were rated as somewhat agree or agree (score = 4), and 0 otherwise. Binary logistic regression analyses were performed to examine whether teacher type (generalist versus PE specialist) predicted the odds of complete agreement within each construct. Gender and years of teaching experience were included as covariates in this model. For all statistical analyses, the statistical significance was determined at *p* < 0.05.

## 4 Results

A total of 228 individuals accessed the online questionnaire. Of these, 24 closed the survey without answering any questions. Of the 204 responses received, 173 teachers (85%) fully completed the questionnaire. Among these 173 teachers, the average age was 35.6 years (±9.78) and 51.4.% was female. Approximately two‐thirds of these participants were PE specialists (70.5%), while the remaining one‐third were generalists (29.5%). The participants' experience in delivering PE lessons varied, with 43.4% having 10 or more years of experience.

### 4.1 Providing Enjoyable PE Lessons

Of the 173 teachers who completed the questionnaire, almost all (*n* = 167 (96.0%)) totally agreed that enjoyment in PE lessons is important. However, fewer reported having a conscious focus on fostering children's PE enjoyment (*n* = 135 totally agreed (78.0%)). No significant differences were found between generalists and PE specialists on either of these measures. Regarding perceived barriers in providing enjoyable PE lessons for every child, six PE specialists indicated that they do not experience any barriers. Among the teachers who perceived barriers (*n* = 167), a significant difference in the proportion of barriers selected across the four categories was found (*χ* ^2^(3) = 33.93, *p* < 0.001). Teacher‐related barriers were selected significantly less often than both facility‐related (adjusted *p* < 0.001) and child‐related barriers (adjusted *p* < 0.001). Child‐related barriers were the most frequently selected (*M* [SD]: 0.34 [±0.33]). This was followed by barriers related to school facilities (*M* [SD]: 0.30 [±0.32]), school policies (*M* [SD]: 0.22 [±0.27]), and teacher‐related barriers (*M* [SD]: 0.14 [±0.24]) (Table 1). Definitions of each barrier category are provided in Table 1 notes a‐d.

**TABLE 1:** Perceived barriers to deliver enjoyable PE lessons, overall and by teacher type.

Category	All teachers	Generalists	PE specialists	Generalist ↔ PE specialist	
	*M* (±SD) *N* = 167	*M* (±SD) *N* = 51	*M* (±SD) *N* = 116	*U*	*p*
Teacher‐related barriers^a^	0.14 (±0.24)	0.25 (±0.30)	0.09 (±0.18)	1992.0	< 0.01*
Child‐related barriers^b^	0.34 (±0.33)	0.16 (±0.26)	0.41 (±0.33)	1615.5	< 0.01*
School‐related barriers: facilities^c^	0.30 (±0.32)	0.29 (±0.33)	0.31 (±0.31)	2844.0	0.68
School‐related barriers: policies^d^	0.22 (±0.27)	0.29 (±0.29)	0.19 (±0.26)	2326.5	0.02*

*Note:* Values represent the mean proportion scores for each main barrier category (range from 0 to 1), where 0 indicates that no barriers were selected in this category, and 1 indicates that all five selected options fell within this category. Bold values represent significance at *p* < 0.05.

Abbreviations: *M* = mean proportion score; SD = standard deviation; U = Mann–Whitney *U* statistic.

^a^ Teacher's knowledge and skills, PE teaching experiences, negative PE experiences, confidence in PE teaching, motor skills, PE lessons preparation, personal interest in PE.

^b^ Children's attitudes toward PE activities, interest in PE, concentration in PE, motivation for PE, perception of the value of PA.

^c^ Location, accommodation, available equipment availability, budget, space for physical activity.

^d^ Schools' emphasis on PE, PE scheduling, PE lesson time, preparation time for PE, access to supportive resources, opportunities for professional development, teacher autonomy in designing PE, teacher autonomy in teaching styles.

Results showed significant differences between PE specialists and generalists for three of the four barrier categories. Compared to PE specialists, generalists were more likely to select teacher‐related barriers (Mann–Whitney *U* = 1992.00; *p* < 0.001), such as inadequate PE lesson preparation and a lack of content expertise, and school policy‐related barriers (Mann–Whitney *U* = 2326.50; *p* = 0.019), like facilitation in lesson preparation time and prioritization of PE by management and colleagues. PE specialists were more likely to select child‐related barriers, such as children's motivation and concentration, than generalists (Mann–Whitney *U* = 4300.50; *p* < 0.001). No significant difference was found between teacher types for school facilities barriers, with similar mean proportion values among generalists and generalists (Mann–Whitney *U* = 3072.00; *p* = 0.682).

### 4.2 Reflective Practices

Teachers expressed high levels of agreement with the constructs “reflection on action,” “reflection with colleagues,” “self‐appraisal,” and “feedback from children” (Table 2), with mean scores ranging from 1.24 (SD = ±0.33) for “reflection on action” to 1.52 (SD = ±0.53) for “feedback from children.” In contrast, teachers showed relatively less agreement with the construct “desire for improvement” (mean (SD): 2.27 (±0.68)). Across all constructs, teacher type did not significantly predict the odds of complete agreement on the constructs, when controlling for gender and years of PE teaching experience. The results of generalists and PE specialists on all constructs were comparable (see Table 3).

**TABLE 2:** Teachers' perspectives on reflective practices, overall and by teacher type.

Construct	All teachers	Generalists	PE specialists
	*M* (±SD) *N* = 173	*M* (±SD) *N* = 51	*M* (±SD) *N* = 122
Reflection on action	1.24 (±0.33)	1.25 (±0.40)	1.24 (±0.30)
Reflection with colleagues	1.43 (±0.46)	1.42 (±0.52)	1.43 (±0.43)
Self‐appraisal	1.53 (±0.45)	1.47 (±0.49)	1.56 (±0.42)
Desire for improvement	2.27 (±0.68)	2.47 (±0.77)	2.18 (±0.62)
Feedback from children	1.52 (±0.53)	1.48 (±0.60)	1.54 (±0.50)

*Note:* Scoring on each construct: 1 (agree) to 4 (disagree).

Abbreviations: *M* = mean; SD = standard deviation.

**TABLE 3:** Binary logistic regression results: Differences in perspectives on reflective practices between generalists and PE specialists.

Construct	*B*	*p*	Exp(*B*)	95% CI for Exp(*B*)
Reflection on action	0.35	0.67	0.67	0.29 to 6.95
Reflection with colleagues	0.23	0.75	1.26	0.31 to 5.07
Self‐appraisal	−0.57	0.19	0.56	0.24 to 1.34
Desire for improvement	−0.15	0.72	0.86	0.37 to 1.97
Feedback from children	−0.63	0.32	0.53	0.16 to 1.83

*Note:* Model was corrected for gender and years of PE teaching experience. Outcome variables were coded as 0 = did not fully agree with one or more items within the construct, 1 = fully agreed on all items within the construct.

Abbreviations: *B* = regression coefficient; Exp(*B*) = odds ratio; 95% CI for Exp(*B*) = 95% confidence interval for the odds ratio.

Overall, 71.7% reported frequently or consistently engaging in reflective practices. Teachers who did not consistently reflect mentioned a lack of time (62.2%), low prioritization (35.6%), and limited awareness (31.9%) as key reasons. No significant differences were found between generalists and PE specialists in either frequency of reflection or perceived barriers. In terms of reflection methods, most teachers reported engaging in internal reflection (92.5%) or informally sharing experiences with (PE) colleagues (71.1%). Methods such as written reflection (8.7%), peer discussions (42.2%), or participation in professional communities (24.9%) were less common. Lesson evaluations were also primarily internal (93.6%), with around half of teachers involving children (64.2%) or using peer feedback (50.3%). Evaluation strategies such as logbooks (9.8%), video analysis (4.0%), or child monitoring systems (21.4%) were less frequently used. PE specialists were significantly more likely than generalists to participate in professional communities (*χ* ^2^(1, *N* = 173) = 11.21, *p* < 0.001), use peer feedback (*χ* ^2^(1, *N* = 167) = 6.51, *p* = 0.01), and use child monitoring systems (*χ* ^2^(1, *N* = 167) = 5.77, *p* = 0.02).

## 5 Discussion

This study explored primary school teachers' perspectives on providing enjoyable physical education lessons and their use of reflective practices to foster PE enjoyment in children. Additionally, it examined potential differences between PE specialists and generalists in these perspectives. Most teachers acknowledged the importance of children's PE enjoyment, but fewer actively focused on fostering enjoyment in their lessons. This discrepancy could be explained by teachers prioritizing skill development and performance over enjoyment, possibly due to a lack of clarity about what fosters PE enjoyment and how to incorporate it into lessons [38].

Consistent with other studies, generalists reported teacher‐related and school policy‐related barriers significantly more often than PE specialists as main barriers to delivering enjoyable PE lessons [39, 40]. These barriers are likely to be attributed to teachers' educational backgrounds and work responsibilities [12, 31]. Generalists received a relatively brief and less specialized PE training compared to PE specialists [12, 31]. Consequently, generalists may feel less confident and competent in providing enjoyable PE [9, 39]. In practice, generalists are predominantly responsible for classroom instruction, limiting their time for PE preparation. Moreover, by providing a PE lesson once a week, their ability for continuous professional development on the job is limited. In contrast, PE specialists reported child‐related factors, such as children's lack of motivation, concentration, and interest, significantly more often than generalists and identified these as their main barriers to delivering enjoyable PE lessons. This reveals a paradox: while PE specialists identify child‐related barriers, they may fail to acknowledge their role and responsibility in fostering a motivational and need‐supportive PE learning environment. This is consistent with the attribution bias, commonly seen in cases of perceived failure [20].

### 5.1 Reflective Practices

In line with previous studies [17, 41], most teachers valued reflective practices. However, a significant negative association was found between years of teaching experience and teachers' desire to improve their PE teaching. More experienced teachers might rely on routines, perceiving little need to change as they view their practices as sufficient [20, 42]. Although many teachers reported engaging in regular reflection, most engaged internally or by sharing experiences with colleagues, rather than through written reflections and reflective discussions with colleagues. This raises concerns about the depth and quality of reflection, as undocumented, internal reflection may be subject to cognitive biases and emotional influences. Areas of improvement might be overlooked, limiting their professional development and, ultimately, PE enjoyment of children [15, 43].

Teachers' undocumented, internal reflection could be explained by perceived time constraints. Reflection requires time and structured approaches [15, 20, 29], yet demanding workloads may hinder its prioritization. Generalists have to manage a broad curriculum, and PE specialists usually teach consecutive classes [39, 44], leaving limited time for reflection [30]. When primary schools fail to allocate time, space, and resources for reflection, teachers may further be discouraged from engaging in reflective practices. Additionally, teachers may lack a conscious focus on reflective practices because they do not fully realize how reflection can enhance the quality of PE teaching or improve children's PE enjoyment. Teachers may lack knowledge or skills to collect meaningful feedback from children, colleagues, or themselves, or they may believe they already understand their children's PE needs [15, 20, 36]. PE specialists were significantly more likely than generalists to engage in reflection through professional networks, which may be very useful for specialists who often work isolated without PE‐specific colleagues. These communities offer opportunities for collaborative learning [29].

### 5.2 Implications for Primary School Teachers

Effective and time‐efficient strategies tailored to the primary PE context are needed to support teachers in engaging in high‐quality reflection. Written, documented reflection including formats such as journals, logbooks, and self‐reflective notes enables teachers to revisit and build on previous insights. Guided templates can enhance the depth of reflection, while digital tools can facilitate documentation, accessibility, and long‐term use [29, 45, 46, 47]. In addition to self‐reflection, teachers should be encouraged to frequently and systematically gather and use feedback from children. Combining teachers' self‐reflection with input from children and colleagues enriches reflection and supports more responsive PE teaching [27, 43, 48]. To make this process feasible, schools should provide time and appropriate tools for reflection without increasing workload [42]. Schools should promote the use of digital reflection tools, given their potential to support the integration of various reflection methods and simplify the documentation and accessibility of reflective practices. While digital tools are increasingly used in secondary and higher education, they remain underutilized in primary PE as a result of context‐inappropriateness [49].

Moreover, tailored strategies seem relevant to improve reflective practices by PE specialists and generalists. Although PE specialists often focus on child‐related challenges, reflecting on their own teaching practices is needed to impact children's enjoyment and motivation in PE. Encouraging pre‐service PE teachers in PE teacher education to engage in self‐assessment and children's feedback could help them recognize their role in fostering enjoyable, need‐supportive environments [14, 29]. Our findings suggest that more years of PE teaching experience are associated with a decreased desire to improve teaching. This further strengthens the need for reflective skills in pre‐service education programs, as it promotes lifelong reflective practices, supports continuous professional development, and enhances the quality of PE lessons [27, 28, 29].

To ensure that generalists feel competent in providing enjoyable PE lessons, schools are encouraged to provide targeted professional development opportunities, such as PE‐specific professional development days and coaching‐on‐the‐job sessions tailored to each teacher's needs and areas of improvement by PE specialists. Collaborative reflection enhances teaching quality and improves teachers' confidence and perceived competence [23, 29, 48]. Finally, to encourage teachers to engage more frequently in high‐quality reflection, its added value should be made more visible. Well‐designed and implemented interventions, potentially shaped by school policy changes, are needed to demonstrate how changes in teachers' reflection practices influence child‐related outcomes, such as enjoyment, engagement, and interest in PE.

### 5.3 Strengths and Limitations

A strength of this study is its exploratory nature, which provides deeper insights into teachers' perspectives on providing enjoyable PE lessons and their reflective practices. Additionally, the specific comparison between PE specialists and generalist teachers provides valuable insights into the unique challenges and practices of each group. The generalizability of the study results is impacted by this differentiation, and the outcomes are particularly relevant for contexts in which PE lessons are taught by both types of teachers. The practical implications, such as tailored strategies for professional development and reflective practices, further highlight the study's contribution to the field.

However, the study has several limitations. The relatively small sample size, the skewed distribution between PE specialists and generalist teachers, although representative for the current Dutch situation (approximately 70% vs. 30%) [50], and potential selection bias based on personal affection with the topic reflection or PE enjoyment warrant cautious interpretation of the findings. Additionally, the use of self‐reported data may have led to over‐ or underestimation of reflection practices [51]. Lastly, while most constructs related to perspectives on reflective practices showed acceptable internal consistency, the reliability of the constructs “self‐appraisal” and “reflection on action” suggests the need for cautious interpretation.

## 6 Conclusions

This study demonstrated that while most teachers recognize the importance of enjoyment in PE, fewer actively focus on fostering it during PE lessons. Generalists identified teacher‐ and school policy‐related barriers as the main obstacles, whereas PE specialists more frequently highlighted child‐related barriers. Both types of teachers frequently used reflective practices, but structured, written reflection and documentation were relatively rare. The main barriers to reflection included a lack of time, insufficient emphasis on reflection, and a lack of conscious focus.

To address these challenges, tailored strategies are recommended to promote reflection and continuous professional development, ultimately enhancing the quality of PE lessons and children's PE enjoyment. PE specialists should be supported in developing reflective practices focused on fostering enjoyment. Generalists may benefit from coaching and targeted professional development in PE‐specific skills. Schools can support this by allocating time and promoting appropriate tools.

## Consent

Written informed consent for data collection, processing, and analysis was obtained from participants at the start of the questionnaire.

## Conflicts of Interest

The authors declare no conflicts of interest.

## Supporting information


**Data S1:** Supporting Information.

## Data Availability

The data that support the findings of this study are available from the corresponding author upon reasonable request.
