# Supplementary material for: “Look at Yourself”: Teachers' Reflective Practices Toward Enjoyment in Primary School Physical Education
Source: J Sch Health. 2025 Oct 7;96(1):e70092. doi: 10.1111/josh.70092 (PMC12669812; doi:10.1111/josh.70092)
Supplement: Supplementary file 1 — Data S1: Supporting Information. [file JOSH-96-0-s001.docx]

Supplementary Material. **Questions assessing teachers’ perspectives on reflective practices.**

|  | **Cronbach’s Alpha** |
| --- | --- |
| **Reflection on action** | **.675** |
| After a PE lesson, I spend time thinking about what was said and done.  After a PE lesson, I think about how things went during the interaction.  After a PE lesson, I wonder about the children’s experience of the PE lesson.  After a PE lesson, I wonder about my own experience of the interaction. | |
| **Reflection with colleagues** | **.804** |
| I find that reflecting with colleagues about how I teach helps me to work out problems I might be having.  When reflecting with colleagues about how I teach I become aware of things I had not previously considered.  I gain new insights when reflecting with colleagues about how I teach.  When reflecting with colleagues about how I teach I develop new perspectives. | |
| **Self-appraisal** | **.633** |
| I think about my weaknesses in teaching.  I think about how I might improve my ability to teach.  I think about my strengths for teaching.  I critically evaluate the strategies and techniques I use when I teach. | |
| **Desire for improvement** | **.756** |
| I think I still have a lot of things to learn to improve my ability to teach.  I desire more experience to improve my ability to teach.  I would like to learn new skills to improve my ability to teach.  I desire more knowledge to improve my ability to teach. | |
| **Feedback from children** | **.795** |
| I feel that children’s feedback is important because it identifies my strengths and weaknesses.  I think children’s feedback is important as it will help me understand them better.  I think it is important to consider children’s feedback, as it will help me enhance my current teaching and perform better in the future. | |
